# 4-Bromo-5-[(2-bromo­ethyl)­sulfanyl]-1,3-dithiole-2-thione

**DOI:** 10.1107/S1600536809036770

**Published:** 2009-09-16

**Authors:** Jing-Jing Ding, Yong-Hua Zhang, Bang-Tun Zhao, Gui-Rong Qu

**Affiliations:** a College of Chemistry and Environmental Science, Henan Normal University, Xinxiang 453002, People’s Republic of China; b College of Chemistry and Chemical Engineering, Luoyang Normal University, Luoyang 471022, People’s Republic of China

## Abstract

The title compound, C_5_H_4_Br_2_S_4_, consists of a statistically planar, 4-bromo-1,3-dithiole-2-thione unit [maximum deviation from the ring plane 0.001 (2) Å], with a bromo­ethyl­sulfanyl substituent in the 5-position. In the crystal structure, weak inter­molecular S⋯S [3.438 (15) and 3.522 (15) Å] and S⋯Br [3.422 (14) and 3.498 (14) Å] inter­actions generate a three-dimensional supra­molecular architecture.

## Related literature

For general background to the applications of halogenated 1,3-dithiole-2-thio­nes, see: Alberola *et al.* 2006 ▶; Batsanov *et al.* (2001 ▶); Jeppesen *et al.* (2004 ▶); Segura & Martin (2001 ▶); Wang *et al.* (1995 ▶). For a related structure, see: Zhao *et al.* (2008 ▶).
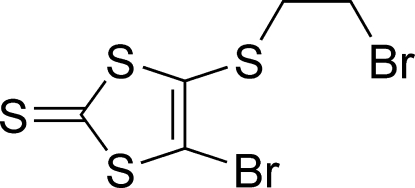


## Experimental

### 

#### Crystal data

C_5_H_4_Br_2_S_4_
*M* *_r_* = 352.14
Monoclinic, 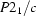

*a* = 4.7892 (12) Å
*b* = 20.381 (5) Å
*c* = 10.809 (3) Å
β = 96.922 (3)°
*V* = 1047.3 (5) Å^3^
*Z* = 4
Mo *K*α radiation
μ = 8.47 mm^−1^
*T* = 294 K
0.44 × 0.17 × 0.06 mm

#### Data collection

Bruker SMART CCD area-detector diffractometer
Absorption correction: multi-scan (*SADABS*; Bruker, 1997 ▶) *T*_min_ = 0.117, *T*_max_ = 0.613
9101 measured reflections
2391 independent reflections
1845 reflections with *I* > 2σ(*I*)
*R*_int_ = 0.033

#### Refinement

*R*[*F* ^2^ > 2σ(*F* ^2^)] = 0.033
*wR*(*F* ^2^) = 0.081
*S* = 1.05
2391 reflections
100 parameters
H-atom parameters constrained
Δρ_max_ = 0.38 e Å^−3^
Δρ_min_ = −0.81 e Å^−3^

### 

Data collection: *SMART* (Bruker, 1997 ▶); cell refinement: *SAINT* (Bruker, 1997 ▶); data reduction: *SAINT*; program(s) used to solve structure: *SHELXS97* (Sheldrick, 2008 ▶); program(s) used to refine structure: *SHELXL97* (Sheldrick, 2008 ▶); molecular graphics: *SHELXTL* (Sheldrick, 2008 ▶); software used to prepare material for publication: *SHELXTL*.

## Supplementary Material

Crystal structure: contains datablocks I, global. DOI: 10.1107/S1600536809036770/sj2640sup1.cif

Structure factors: contains datablocks I. DOI: 10.1107/S1600536809036770/sj2640Isup2.hkl

Additional supplementary materials:  crystallographic information; 3D view; checkCIF report

## Figures and Tables

**Fig. 1.**
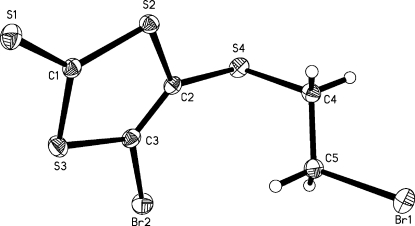
The molecular structure of the title compound with ellipsoids drawn at the 30% probability level.

**Fig. 2.**
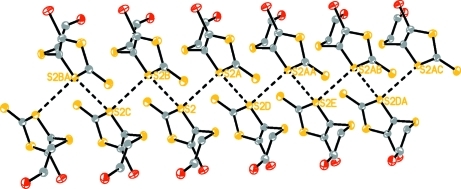
The 1-dimensional chain formed by S···S interactions, shown as dashed lines.

**Fig. 3.**
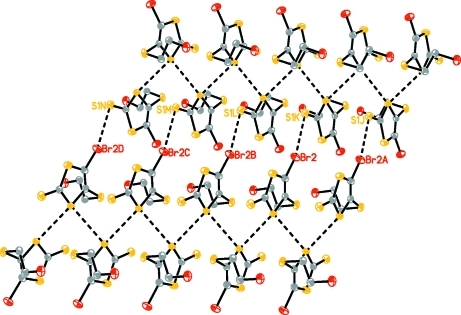
The 2-dimensional sheet formed by intermolecular S2···S2 and S1···Br2 interactions, shown as dashed lines.

**Fig. 4.**
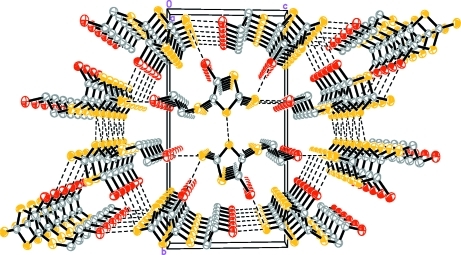
The 3-dimensional network formed by intermolecular S2···S2, S1···Br2 and S1···Br1 interactions, shown as dashed lines.

## supplementary crystallographic information

### Comment

Tetrathiafulvalene (TTF) and its derivatives have attracted great interest for their high electronic conductivity, superconductivity as well as supramolecular features (Segura & Martin, 2001; Jeppesen *et al.*, 2004). The attachment of halogen atoms to TTF framework reduces the π-electron donating ability and this effect is additive with an increasing number of halogens on the TTF system (Wang *et al.*, 1995), As important precursors to the halogenated TTF derivatives, 1,3-dithiole-2-(thi)ones involving bromine groups have also attracted attention (Batsanov *et al.*, 2001; Alberola *et al.*, 2006). We describe here the synthesis and structure of a novel 4-bromo-5-[(2-bromoethyl)sulfanyl]-1,3-dithiole-2-thione compound, (I) (Fig. 1).

As seen from Fig. 1, all five atoms of five-membered dithiole ring and three  exocyclic S1, Br1 and S4 atoms are nearly coplanar with a maximum deviation from the least-squares plane of only 0.1045 Å (Br2). The C-S bond lengths range from 1.647 (4) to 1.814 (4) Å. The bond distances C1-S1 (1.647 (3)) Å, C2-S4 (1.753 (3)) Å, and Br2-C3(1.883 (4)) Å  are relatively short which indicates a degree of conjugation of the S1, S4 and Br2 substituents with the 1,3-dithiol ring system. However, the C4-S4 bond is typical of a single bond with a bond length of 1.814 (4) Å. The structure of title compound is very similar to that of 3-(2-thioxo-1,3- dithiol-4-ylsulfanyl)propanenitrile (Zhao *et al.*, 2008).

In the crystal structure, molecules of (I) form 1-dimensional chains by way of intermolecular S···S interactions along *a* axis (Fig.2). The distances between alternate S2 atoms are 3.438 (15) Å and 3.522 (15) Å, respectively. In addition, the 1-dimensional chains are interconnected by intermolecular S1···Br2 interactions (S1···Br2 = 3.422 (14) Å) to generate a 2-dimensional sheet (Fig. 3) in the *ab* plane. These are further linked by intermolecular Br1···S1 interactions (S1···Br1 = 3.498 (14) Å) to form a 3-dimensional supramolecular structure (Fig. 4).

### Experimental

A solution of PPh_3_(3.04 g, 11.6 mmol) in dichloromethane (20 mL) was added dropwise to a solution of 4-(2-hydroxyethylsulfanyl)-1,3-dithiole-2-thione (1.67 g, 11.6 mmol) and CBr_4_ (3.84 g, 11.6 mmol), also in dichloromethane (50 mL), over 2 h. The mixture was then stirred for 8 h at room temperature. The resulting solution was washed with water and dried with Na_2_SO_4_. The solvent was then evaporated under reduced pressure and the crude product was purified by column chromatography on silica. (dichloromethane:petroleum ether= 2:3) to yield the title compound as yellow solid in 85 % yield. Yellow block-like single crystals were obtained from slow evaporation of a dichloromethane solution at room temperature.

### Refinement

All H-atoms were positioned geometrically and refined using a riding model with d(C-H) = 0.97 Å, U_iso_ = 1.2U_eq_ (C) for CH_2_ atoms.

### Crystal data

**Table:**

C_5_H_4_Br_2_S_4_	*F*(000) = 672
*M**_r_* = 352.14	*D*_x_ = 2.233 Mg m^−^^3^
Monoclinic, *P*2_1_/*c*	Melting point: 331 K
Hall symbol: -P 2ybc	Mo *K*α radiation, λ = 0.71073 Å
*a* = 4.7892 (12) Å	Cell parameters from 3116 reflections
*b* = 20.381 (5) Å	θ = 3.6–26.1°
*c* = 10.809 (3) Å	µ = 8.47 mm^−^^1^
β = 96.922 (3)°	*T* = 294 K
*V* = 1047.3 (5) Å^3^	Block, yellow
*Z* = 4	0.44 × 0.17 × 0.06 mm

### Data collection

**Table:**

Bruker SMART CCD area-detector diffractometer	2391 independent reflections
Radiation source: fine-focus sealed tube	1845 reflections with *I* > 2σ(*I*)
graphite	*R*_int_ = 0.033
φ and ω scans	θ_max_ = 27.5°, θ_min_ = 2.8°
Absorption correction: multi-scan (*SADABS*; Bruker, 1997)	*h* = −6→6
*T*_min_ = 0.117, *T*_max_ = 0.613	*k* = −26→26
9101 measured reflections	*l* = −13→14

### Refinement

**Table:**

Refinement on *F*^2^	Primary atom site location: structure-invariant direct methods
Least-squares matrix: full	Secondary atom site location: difference Fourier map
*R*[*F*^2^ > 2σ(*F*^2^)] = 0.033	Hydrogen site location: inferred from neighbouring sites
*wR*(*F*^2^) = 0.081	H-atom parameters constrained
*S* = 1.05	*w* = 1/[σ^2^(*F*_o_^2^) + (0.0361*P*)^2^ + 0.5713*P*] where *P* = (*F*_o_^2^ + 2*F*_c_^2^)/3
2391 reflections	(Δ/σ)_max_ = 0.001
100 parameters	Δρ_max_ = 0.38 e Å^−^^3^
0 restraints	Δρ_min_ = −0.81 e Å^−^^3^

### Special details

**Table:**

Geometry. All esds (except the esd in the dihedral angle between two l.s. planes) are estimated using the full covariance matrix. The cell esds are taken into account individually in the estimation of esds in distances, angles and torsion angles; correlations between esds in cell parameters are only used when they are defined by crystal symmetry. An approximate (isotropic) treatment of cell esds is used for estimating esds involving l.s. planes.
Refinement. Refinement of F^2^ against ALL reflections. The weighted R-factor wR and goodness of fit S are based on F^2^, conventional R-factors R are based on F, with F set to zero for negative F^2^. The threshold expression of F^2^ > 2sigma(F^2^) is used only for calculating R-factors(gt) etc. and is not relevant to the choice of reflections for refinement. R-factors based on F^2^ are statistically about twice as large as those based on F, and R- factors based on ALL data will be even larger.

### Fractional atomic coordinates and isotropic or equivalent isotropic displacement parameters (Å^2^)

**Table:**

	*x*	*y*	*z*	*U*_iso_*/*U*_eq_	
Br1	0.33646 (9)	0.39616 (2)	−0.03831 (4)	0.05782 (14)	
Br2	−0.12031 (10)	0.252098 (19)	0.33275 (4)	0.06158 (15)	
S1	0.6869 (2)	0.39508 (5)	0.69591 (9)	0.0506 (2)	
S2	0.25370 (18)	0.43827 (4)	0.49022 (8)	0.0406 (2)	
S3	0.3149 (2)	0.30064 (4)	0.54490 (10)	0.0517 (3)	
S4	−0.20103 (18)	0.42118 (5)	0.27552 (9)	0.0481 (2)	
C1	0.4319 (7)	0.37956 (16)	0.5834 (3)	0.0379 (7)	
C2	0.0385 (7)	0.38613 (16)	0.3924 (3)	0.0379 (7)	
C3	0.0692 (8)	0.32205 (17)	0.4204 (3)	0.0439 (8)	
C4	0.0261 (7)	0.43820 (17)	0.1567 (3)	0.0432 (8)	
H4A	−0.0737	0.4654	0.0923	0.052*	
H4B	0.1900	0.4623	0.1935	0.052*	
C5	0.1193 (8)	0.37586 (17)	0.0993 (4)	0.0458 (8)	
H5A	−0.0441	0.3498	0.0686	0.055*	
H5B	0.2340	0.3503	0.1620	0.055*	

### Atomic displacement parameters (Å^2^)

**Table:**

	*U*^11^	*U*^22^	*U*^33^	*U*^12^	*U*^13^	*U*^23^
Br1	0.0521 (2)	0.0758 (3)	0.0462 (3)	0.00042 (19)	0.00845 (17)	0.00156 (19)
Br2	0.0753 (3)	0.0501 (2)	0.0572 (3)	−0.01945 (19)	−0.0007 (2)	−0.00821 (18)
S1	0.0503 (5)	0.0586 (6)	0.0406 (5)	−0.0006 (4)	−0.0045 (4)	0.0014 (4)
S2	0.0410 (5)	0.0369 (4)	0.0424 (5)	−0.0004 (3)	−0.0003 (4)	−0.0019 (3)
S3	0.0659 (6)	0.0388 (4)	0.0483 (6)	−0.0032 (4)	−0.0021 (5)	0.0048 (4)
S4	0.0341 (5)	0.0628 (5)	0.0462 (5)	0.0083 (4)	0.0001 (4)	−0.0038 (4)
C1	0.0390 (18)	0.0420 (17)	0.0342 (19)	0.0010 (14)	0.0098 (14)	0.0002 (14)
C2	0.0346 (17)	0.0441 (17)	0.0349 (19)	−0.0009 (14)	0.0039 (13)	−0.0031 (14)
C3	0.048 (2)	0.0448 (18)	0.039 (2)	−0.0081 (16)	0.0068 (16)	−0.0056 (15)
C4	0.0414 (19)	0.0425 (18)	0.043 (2)	0.0030 (15)	−0.0038 (15)	0.0016 (15)
C5	0.045 (2)	0.0448 (18)	0.048 (2)	−0.0022 (15)	0.0083 (16)	−0.0004 (16)

### Geometric parameters (Å, °)

**Table:**

Br1—C5	1.959 (4)	S4—C4	1.814 (4)
Br2—C3	1.883 (3)	C2—C3	1.345 (5)
S1—C1	1.647 (4)	C4—C5	1.505 (5)
S2—C1	1.723 (3)	C4—H4A	0.9700
S2—C2	1.746 (3)	C4—H4B	0.9700
S3—C3	1.734 (4)	C5—H5A	0.9700
S3—C1	1.737 (3)	C5—H5B	0.9700
S4—C2	1.753 (4)		
			
C1—S2—C2	98.40 (16)	C5—C4—S4	111.3 (2)
C3—S3—C1	97.02 (17)	C5—C4—H4A	109.4
C2—S4—C4	101.05 (16)	S4—C4—H4A	109.4
S1—C1—S2	124.7 (2)	C5—C4—H4B	109.4
S1—C1—S3	122.9 (2)	S4—C4—H4B	109.4
S2—C1—S3	112.34 (19)	H4A—C4—H4B	108.0
C3—C2—S2	114.5 (3)	C4—C5—Br1	110.2 (2)
C3—C2—S4	127.0 (3)	C4—C5—H5A	109.6
S2—C2—S4	118.41 (19)	Br1—C5—H5A	109.6
C2—C3—S3	117.7 (3)	C4—C5—H5B	109.6
C2—C3—Br2	126.1 (3)	Br1—C5—H5B	109.6
S3—C3—Br2	116.2 (2)	H5A—C5—H5B	108.1
			
C2—S2—C1—S1	177.1 (2)	S2—C2—C3—S3	−0.9 (4)
C2—S2—C1—S3	−2.4 (2)	S4—C2—C3—S3	−177.7 (2)
C3—S3—C1—S1	−177.5 (2)	S2—C2—C3—Br2	−178.3 (2)
C3—S3—C1—S2	2.0 (2)	S4—C2—C3—Br2	4.9 (5)
C1—S2—C2—C3	2.0 (3)	C1—S3—C3—C2	−0.7 (3)
C1—S2—C2—S4	179.1 (2)	C1—S3—C3—Br2	176.9 (2)
C4—S4—C2—C3	−102.6 (3)	C2—S4—C4—C5	69.7 (3)
C4—S4—C2—S2	80.7 (2)	S4—C4—C5—Br1	175.02 (17)

## References

[bb1] Alberola, A., Collis, R. J., Garcia, F. & Howard, R. E. (2006). *Tetrahedron*, **62**, 8152–8157.

[bb2] Batsanov, A. S., Bryce, M. R., Chesney, A., Howard, J. A. K., John, D. E., Moore, A. J., Wood, C. L., Gershtenman, H., Becker, J. Y., Khodorkovsky, V. Y., Ellern, A., Bernstein, J., Perepichka, I. F., Rotello, V., Gray, M. & Cuello, A. O. (2001). *J. Mater. Chem.* **11**, 2181–2191.

[bb3] Bruker (1997). *SMART*, *SAINT* and *SADABS* Bruker AXS Inc., Madison, Wisconsin, USA.

[bb4] Jeppesen, J. O., Nielsen, M. B. & Becher, J. (2004). *Chem. Rev.* **104**, 5115–5131. 10.1021/cr030630u 15535644

[bb5] Segura, J. L. & Martin, N. (2001). *Angew. Chem. Int. Ed.* **40**, 1372–1409. 10.1002/1521-3773(20010417)40:8<1372::aid-anie1372>3.0.co;2-i 11317287

[bb6] Sheldrick, G. M. (2008). *Acta Cryst.* A**64**, 112–122. 10.1107/S0108767307043930 18156677

[bb7] Wang, C., Becker, J. Y., Bernstein, J., Ellern, A. & Khodorkovsky, V. (1995). *J. Mater. Chem.* **5**, 1559–1562.

[bb8] Zhao, B.-T., Ding, J.-J. & Qu, G.-R. (2008). *Acta Cryst.* **E64**, o2078. 10.1107/S1600536808031711 PMC2959697 21580943

